# Artificial switches induce the bespoke production of functional compounds in marine microalgae *Chlorella* by neutralizing CO_2_

**DOI:** 10.1186/s13068-023-02381-5

**Published:** 2023-09-27

**Authors:** Jiahua Gu, Yuan Xiao, Mingcan Wu, Aoqi Wang, Xinyu Cui, Yi Xin, Kalyanee Paithoonrangsarid, Yandu Lu

**Affiliations:** 1 Single-cell BioEngineering Group, State Key Laboratory of Marine Resource Utilization in South China Sea, School of Marine Biology and Fisheries, Hainan University, Haikou, 570228 China; 2 Biochemical Engineering and Systems Biology Research Group, National Center for Genetic Engineering and Biotechnology, National Science and Technology Development Agency, King Mongkut’s University of Technology Thonburi, Bangkok, Thailand; 3 Hainan Provincial Key Laboratory of Tropical Hydrobiotechnology, Hainan University, Haikou, China; 4 Haikou Technology Innovation Center for Research and Utilization of Algal Bioresources, Hainan University, Haikou, China

**Keywords:** *Chlorella*, CO_2_ tolerance, Microalgae, Multiomics, Mutant library, Lipids

## Abstract

To improve the CO_2_ tolerance of a marine microalga *Chlorella* sp. of which the production capacity has been demonstrated industrially, a mutant library was created and a strain *hct53* was screened. Compared to the parental strain, *hct53* shows a high CO_2_ capture capacity, while starch biosynthesis is compromised, with increases in health beneficial metabolites and antioxidant capacity. Global gene expression and genome-wide mutation distribution revealed that transcript choreography was concomitant with more active CO_2_ sequestration, an increase in the lipid synthesis, and a decrease in the starch and protein synthesis. These results suggest that artificial trait improvement via mutagenesis, couple with multiomics analysis, helps discover genetic switches that induce the bespoke conversion of carbon flow from “redundant metabolites” to valuable ones for functional food.

**Supplementary Information:**

The online version contains supplementary material available at 10.1186/s13068-023-02381-5.

## Introduction

Algae sequester carbon dioxide (CO_2_) and convert it into photosynthate, which could be explored for human ends ranging from drugs to functional foods [1, 2]. Microalgae are the potential choice in diverting carbon emission from industrial plants. Owing to the high CO_2_ concentrations, industrial flue gas can constrain the growth of most microalgae. Therefore, creation of high-CO_2_-tolerant microalgal strains to feed on CO_2_-rich industrial flue gas would display mutual benefits as an ideal way to sequester CO_2_ and produce versatile value-added compounds.

Sugars, lipids, and proteins are primary metabolites in microalgae [3]. Among them, polyunsaturated fatty acids (PUFAs) are essential nutrients, because they cannot be synthesized by humans. PUFAs from microalgae are high-value nutraceuticals and have been widely used as additives in human diets and baby formula due to their benefits for human health, such as promoting cognitive and visual development of infants, reducing the risk of cardiovascular diseases, and preventing age-related disorders [4, 5]. Thus, humans must obtain them from the dietary supplements.

In contrast, as the major component of polysaccharides in most microalgae, starch serves as energy storage and has minor pharmaceutical effects in humans. Plenty of commercial starch is available from corn, wheat, tapioca, and potato [6]. On the other hand, sulfated polysaccharides (SPs) possess a variety of biological activities, such as anticoagulant, antiviral and immuno-inflammatory, antilipidemic, and antioxidant activities [7–9]. However, there is still a need to discover genetic switches that induce the bespoke conversion of carbon flow from ‘redundant’ metabolites to valuable ones and artificially create ‘super’ microalgal strains that capture industrial flue gas CO_2_ and produce pharmaceutically and nutritionally active compounds for human beings.

While *Chlorella* spp. have been cultivated for their high protein and carotenoid content, there is still a need to improve the production of other valuable compounds in microalgae. With the growing demand for alternative sources of fresh water [10], the use of marine microalgae in industrial systems is particularly attractive, as they can grow on non-arable land and utilize saline water supplies. By discovering genetic switches that can direct carbon flow towards the synthesis of desired compounds, such as PUFAs and SPs, ‘super’ microalgal strains could be created that capture industrial flue gas CO_2_ and produce customized compounds for human consumption.

We previously isolated a marine *Chlorella* strain MEM25 (hereafter MEM25), which has demonstrated a remarkable ability to grow under various environmental conditions, including extreme weather [11]. In addition to its fast growth rate, MEM25 produces high amounts of carotenoids and protein, making it a promising candidate for industrial-scale cultivation [11]. It produces high amounts of valuable metabolites, particularly carotenoids [12] and proteins (>50% of dry weight, DW) [11]. To realize the close-loop production of valuable bioproducts and achieve sustainable, low-carbon, and circular bioeconomy, MEM25 has been employed as a model to probe its capacity in restoration of high-salinity seafood processing wastewater and production of value-added compounds for functional food (Chen et al., *unpublished*). However, the profitability of customized-product systems by precisely control the carbon flow in MEM25 has not been appreciated. However, To maximize the economic potential of MEM25, its carbon flow needs to be precisely controlled to produce desired metabolites. To improve the food feature of MEM25, we established an engineering system which further facilitate it as a cell factory [https://pubmed.ncbi.nlm.nih.gov/37679828/]. While genetic engineering of model microalgae species has been successful in manipulating metabolic pathways, this approach requires sophisticated techniques and raises ethical concerns [13–17]. Alternatively, chemical and physical mutagens offer effective ways to generate genetic variations and create new strains with desired phenotypes, without the need for genetic modification (non-GMOs). Therefore, we believe that combining traditional mutagenesis approaches with advanced know-how methods could provide a feasible solution for developing customized-product systems using MEM25.

Therefore, in this study, we conducted a study using *Chlorella* sp. MEM25 as a model to improve its biotechnological properties. We employed EMS-mediated mutagenesis breeding, which resulted in a mutant population. From the population, a strain called *hct53* was screened for desirable traits (high CO_2_ capture capacity and customized production of both UFAs and SPs). We evaluated *hct53*’s potential as a source of food ingredients and found it to be promising. To elucidate the genetic mechanisms underlying these traits, we performed global gene expression analysis and genome-wide mutation distribution mapping. Our findings suggest that it is possible to artificially induce the production of functional molecules in microalgae by neutralizing CO_2_ and improve their nutritional value as food additives. This study provides a valuable approach for developing microalgal strains with tailored properties through non-GMO mutagenesis breeding.

## Materials and methods

### Algal strains and culture conditions

*Chlorella* sp. MEM25 (MEM25) was preserved in Single-cell BioEngineering Group, State Key Laboratory of Marine Resource Utilization in South China Sea, Hainan University. The strain is typically cultivated in enriched F2 cultures with a salinity of 35‰, at an ambient temperature of 25 °C, under light intensities of 50 μmol·photons·m^−2^ s^−1^ [11].

### Mutagenesis and screening high CO_2_ tolerance

To generate a mutant pool of MEM25, we treated log-phase cells with different concentrations of EMS for varying durations. The cells were then centrifuged, and the reaction was stopped by adding 10% (w/v) Na_2_S_2_O_3_. The  cells were collected, washed twice with PBS, and resuspended in fresh F2 medium at a concentration of 1.0 × 10^4^ cells·mL^−1^. Subsequently, 100 μL of the algal suspension was plated on solid F2 and cultivated under dim light conditions for 2 weeks. We optimized the mutagenesis conditions by assessing the number and morphology of the resulting colonies.

We constructed a mutant pool using the optimized conditions and further assessed candidate mutants for growth in an CO_2_ incubator under continuous light (LRH-250-TE, Hongjun Instrument Technology Co., Ltd. China). The cells’ growth was monitored by Microplate Reader (Infnite^®^ E Plex, Tecan). Ten candidate mutants were assessed further for growth in an CO_2_ incubator with 5% CO_2_ (v/v) and continuous light at 50 μmol·photons·m^−2^ s^−1^. The cell density, cell size, and pigment content were measured at the indicated day.

### Measurement of cell density, cell size, and pH values

To monitor the growth of microalgae, cell density and cell size were measured using a Luna FL automated fluorescence cell counter (Logos Biosystems, Korea). The pH values of algal culture were determined by a FiveEasy Plus pH meter (Mettler-Toledo, Switzerland).

### Determination of pigment content

The contents of chlorophylls and carotenoids were determined following a previously described method with modifications [13, 18]. In brief, 1 mL algal culture was centrifuged (12,000*g* for 3 min) and the supernatant was disposed. Cell pellets were resuspended in 1 mL methanol, bead beat with glass beads for 1 min twice, followed by incubation in the dark at 60 °C for 15 min. Next, the mixture was centrifuged at 12,000*g* for 15 min to remove cellular debris and the resulting supernatant was used to determine the pigment contents by spectrophotometer measurement (GeneQuantTM 1300, GE). The contents of chlorophylls and carotenoids were calculated using Eqs. (1–3):

1 $${\text{Chlorophyll a }} = { 13}.{9}\left( {{\text{OD}}_{{665}} - {\text{ OD}}_{{75}0} } \right)$$

2 $${\text{Chlorophyll b }} = { 21}.{\text{43 OD}}_{{644}} - { 4}.{\text{65 OD}}_{{662}}$$

3 $${\text{Carotenoids }} = { 4}.{\text{7A}}_{{44}0} - \, \left( {{1}.{\text{38OD}}_{{662}} + { 5}.{\text{48OD}}_{{644}} } \right)$$

### Metabolic analysis

Algal cells were cultured in a 300-mL photobioreactor under nitrogen-replete (+N), low light (LL, 50 μmol·photons·m^−2^ s^−1^) conditions and nitrogen-depleted (1/16N, −N), high light (HL, 200 μmol·photons·m^−2^ s^−1^) conditions, with 0.04% (air) and 5% (v/v) CO_2_. The total lipids and fatty acids were measured as our earlier study [13]. The total carbohydrates content was measured by the phenol–sulfuric acid method [19]. The starch content was measured using Total Starch Kit (K-TSTA-1107, Megazyme, Ireland) [20]. We determined the non-starch carbohydrate (NSC) content by subtracting the starch content from the total carbohydrates content.

### Determination of monosaccharide and uronic acid contents

The monosaccharide and uronic acid contents were measured according to the previous studies [21, 22]. Briefly, 10 mg of algal powder was hydrolyzed in a sealed tube with 2 M trifluoroacetic acid (TFA) at 100 ℃ for 6 h. Then, 500 μL of the standard solutions containing each monosaccharide (mannose, rhamnose, glucuronic acid, galacturonic acid, glucose, galactose, arabinose, or fucose) and the hydrolysate, were filtered through a 0.22 μm membrane filter, and transferred to the tube. Next, 500 μL of 0.3 mol/L NaOH solution was added, followed by 500 μL of 0.5 mol L^−1^ 1-phenyl-3-methyl-5-pyrazolone (PMP) solution (with methanol as the solvent).

After cooling, 500 μL of 0.3 mol L^−1^ HCl solution was added to neutralize the NaOH, and 1 mL of chloroform was added. We then centrifuged the mixture, removed the chloroform layer and repeated the extraction two more times to remove excess PMP. Finally, the sample was filtered through a 0.22 μm membrane filter and analyzed by high-performance liquid chromatography (HPLC; Agilent 1260 Infinity, USA).

### Determination of sulfate content and molecular weight

The sulfate content was measured by the barium chloride–gelatin method. To determine the molecular weights of the total carbohydrates, we used an HPLC system (Waters 515 GPC) at 35 ℃ with a flow rate of 0.5 mL/min using a sodium NaN_3_ solution (0.7%) as the mobile phase. An ultrahydrogel 120 Column coupled to 500 Column and a 2140 Refractive Index Detector (RID) were used. Glycans with a series of known molecular weights were used to prepare a standard curve.

### Fourier transform infrared analysis

An amount of 2 mg of total carbohydrates of the *hct53* mutant and WT was used for the analysis using an FTIR spectrometer (Bruker, Karlsruhe, Germany). The spectra were recorded over the wavelength rang of 500–4000 cm^−1^ with a resolution of 4 cm^−1^ 32 scans. We measured several properties of the total carbohydrates, including monosaccharide composition, uronic acid content, sulfate content, and molecular weight.

### Determination of antioxidants

Antioxidants were determined by assessing the scavenging capacity against α, α-diphenyl-β-picrylhydrazyl (DPPH) [23]. Briefly, 1 mL of DPPH solution (0.1 mM DPPH in 50% ethanol solution) was incubated with a gradient of samples. The reaction mixture was shaken and incubated for 20 min at room temperature. The absorbance of the solution was then measured at 517 nm. The radical scavenging activity was calculated using the following equation:

4 $${\text{Scavenging}}\,{\text{effect}}\,(\% ) = \frac{{1 - {\text{OD}}517}}{{{\text{OD}}517}} \times 100\%$$

### Determination of total lipid and fatty acid contents

A minimum of 10 mg algal powder was transferred to a 2 mL glass bottle (Agilent, USA). Chloroform methanol solution (20/10, v/v) was added, followed by overnight shaking. Afterwards, 0.5 mL KCl solution (0.7%) was added, followed by a centrifugation at 1000 rpm for 10 min. Next, 300 μL of the chloroform layer from the bottom was transferred to a pre-weighed Agilent bottle (*Wb*). The sample was dried with nitrogen, and stored at − 80 ℃ for 20 min, followed by desiccation using an LGJ-12A vacuum freeze dryer (Beijing Sihuan Qihang Technology Co., Ltd, China) for 2 h. The bottle was weighed (*Wa*), and the total lipid content (%) was calculated using the following equation:

5 $${\text{Total}}\,{\text{lipid}}\,{\text{content}}\,(\% {\text{DW}}) = \frac{Wa - Wb}{m} \div 0.45 \times 100\%$$

After calculating the total lipid content, the sample was reconstituted with a chloroform methanol solution (1:1, v:v) and the fatty acid content was determined using GC–MS (8860-5977b; Agilent, USA) following a method described in our previous study [24].

### Transcriptome sampling and sequencing

Mid-logarithmic phase algal cells were transferred into darkness overnight, followed by exposure to high light and nitrogen-depleted conditions with 5% CO_2_. After 96 h, aliquots of cells were collected for transcript analysis. The total RNA of the algal cells was prepared using an RNA miniprep kit (CWBIO) and the quantity and purity were analyzed using the Bioanalyzer 2100 and RNA 1000 Nano LabChip Kit (Agilent, USA), with an RNA integrity value of > 7.0. Poly(A) RNA was purified from 5 μg of total RNA using poly-T oligo-attached magnetic beads with two rounds of purification.

After purification, the mRNA was fragmented into small pieces with divalent cations at an elevated temperature. We then reverse-transcribed the cleaved RNA fragments to create the final complementary DNA (cDNA) library, following the protocol of the mRNA Seq Sample Preparation Kit (Illumina, USA). The average insert size for the paired-end libraries was 300 bp (± 50 bp). Finally, paired-end sequencing was performed on the Illumina Novaseq™ 6000 platform.

### De novo assembly, UniGene annotation, and functional classification

To remove the reads that contained adaptor contamination, low-quality bases, and undetermined bases, we performed read trimming using Cutadapt and Perl scripts [25]. Subsequently, sequence quality was verified using FastQC (http://www.bioinformatics.babraham.ac.uk/projects/fastqc/), including the Q20, Q30, and GC content of the resulting clean data. All downstream analyses were based on high-quality clean data. For de novo assembly of the transcriptome, we used Trinity version 2.4.0 [26]. The longest transcript in the cluster was selected as the gene sequence (i.e., UniGene). All assembled UniGenes were aligned against the nonredundant protein database (http://www.ncbi.nlm.nih.gov/), the Gene Ontology (GO) database (http://www.geneontology.org), Swiss-Prot (http://www.expasy.ch/sprot/), KEGG (http://www.genome.jp/kegg/), and EggNOG (http://eggnogdb.embl.de/) databases using DIAMOND [27] with an e value threshold of <10^–5^.

### Estimation of differential gene expression

To measure gene expression in the mRNA-Seq data sets under each experimental condition, we used Cufflinks (version 2.0.4) to quantify the numbers of aligned reads to annotated genes. We also used Salmon [28] to estimate the expression levels of UniGenes by calculating the transcripts per kilobase million (TPM) [29]. The differentially expressed UniGenes with log_2_ (fold change) greater than 1 or less than − 1, and with statistical significance (*p* value < 0.05), were selected [30].

### Computational pipeline for identifying mutations

To identify mutations, we constructed a computational pipeline using SnpEff [31]. The raw reads of each mutant line were aligned to MEM25 reference genome using default parameters. To reduce false positive calls of mutations, we removed reads that mapped to multiple locations in the genome and retained only uniquely mapped reads for downstream analyses. This was done using the Genome Analysis Toolkit (GATK) [32]. Genotype likelihoods and genotype calls was generated in a VCF file that contained all EMS mutant lines. Using this approach, we could detect only mutations that were unique to one EMS line.

### Statistical analysis

Samples were analyzed in triplicate and the averages and standard deviations were calculated. To assess the differences between paired groups, we used repeated-measures one- or two-way analysis of variance (ANOVA) followed by pairwise comparison with Sidak’s multiple-comparisons test. GraphPad Prism version 9.3 was used to perform statistical analyses and construct figures.

## Results and discussion

### High-throughput creation of a mutant library and selection of mutants with high CO_2_ tolerance

To improve the CO_2_ capture capacity of *Chlorella*, parental stain MEM25 was exposed to EMS at a serial of doses (0.5%, 1%, 1.5%, and 2%) for 1, 2, and 4 h. The lethality of the EMS treatment on MEM25 was assessed. We selected the 1% EMS treatment for 4 h, because it achieved a mortality range of 15–50%, which could result in high numbers of nonsense mutations [24]. MEM25 cells in midlogarithmic phase were transferred to F2 agar plates following the 1% EMS treatment for 4 h, and these plates were incubated at 25 °C for 15 days. The growth was scored visually and more than 35,000 colonies were obtained.

**Fig. 1:**
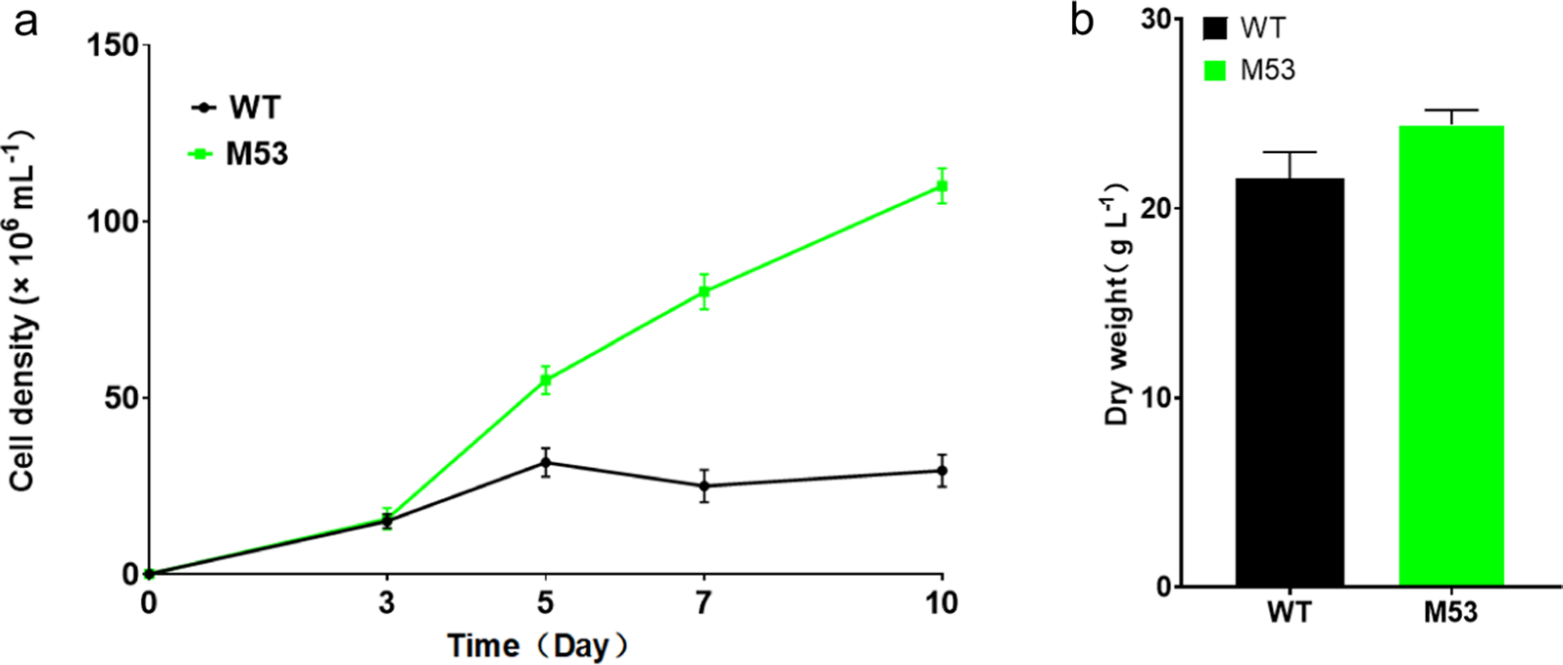
Growth performance of the *high-CO*_*2*_*-tolerance 53* (*hct53*) mutant under high CO_2_ (5%) conditions. **a** cell number; **b** dry cell weight at the end of detection

After preliminary evaluation of growth behavior under ambient atmosphere, ten mutants, displaying a relatively similar or more rapid growth compared with the wild-type algae (WT), were further investigated for their growth under high-concentration of CO_2_ (i.e., 5%). Under high-CO_2_ conditions, mutant strain M53 demonstrated significantly better growth than WT, with increased cell densities (Fig. 1a) and biomass (Fig. 1b). Thus, we designated the strain M53 as *high-CO*_*2*_*-tolerance 53* (*hct53*) for further investigations.

### The ***hct53*** mutant shows a high CO_2_ capture capacity under either low- or high-CO_2_ conditions

**Fig. 2:**
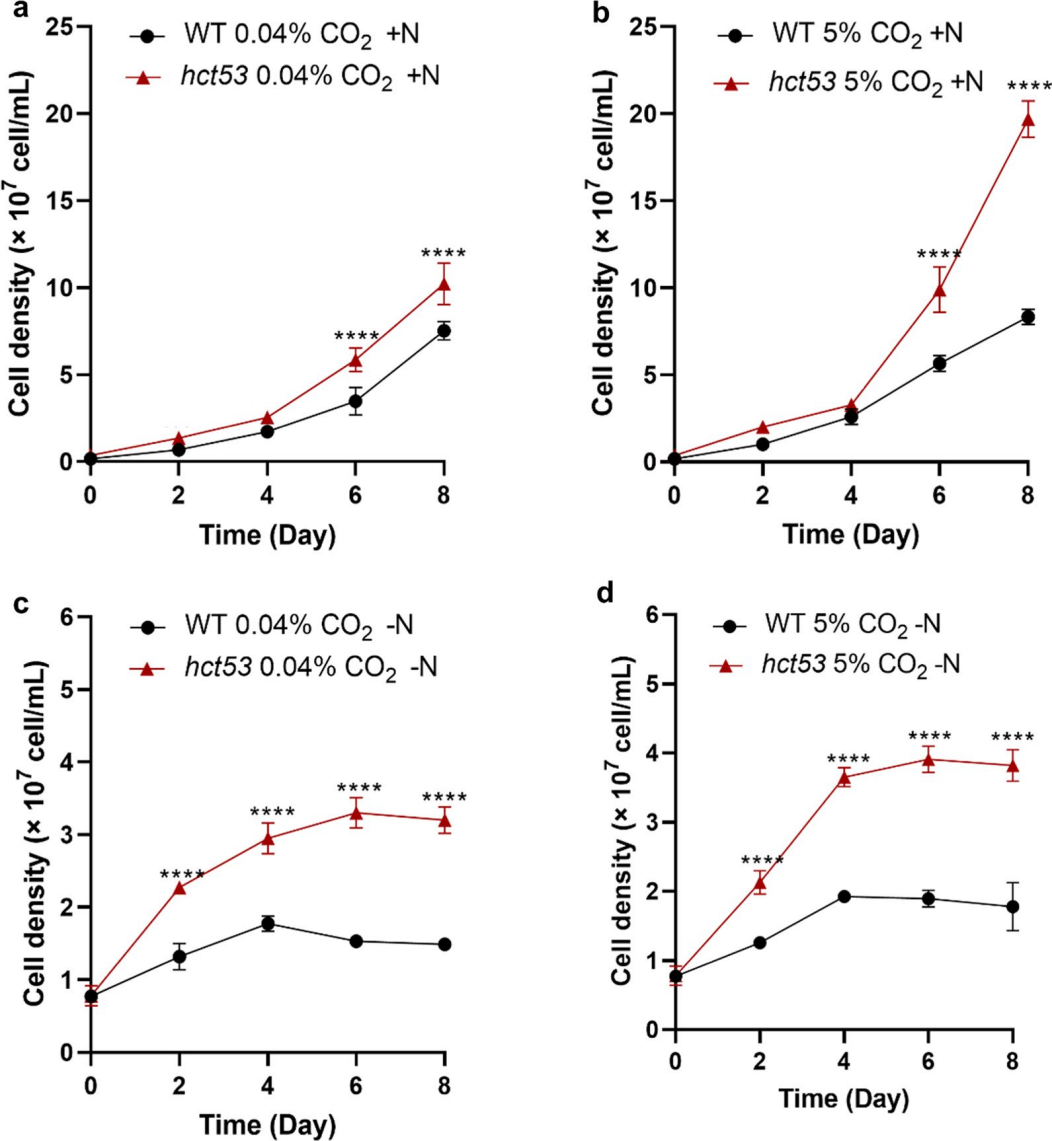
Comparison between the growth of WT and the *hct53* mutant under different conditions. **a** +N conditions aerated with 0.04% CO_2_. **b** +N conditions aerated with 5% CO_2_. **c** −N conditions aerated with 0.04% CO_2_. **d** −N conditions aerated with 5% CO_2_. Abbreviations: WT, wild type; +N, nitrogen-replete conditions; −N, nitrogen-depleted conditions. ****p < 0.0001. The microalgae were cultured in 100-mL column bubbling with 0.04% or 5% CO_2_, respectively

The mutant strain *hct53* demonstrated a higher growth rate than WT when cultured with either 0.04% or 5% CO_2_ (Fig. 2). Under nitrogen-replete conditions (+N), the cell density of *hct53* (1.02 × 10^8^ cell·mL^−1^) was 36.9% higher than that of WT (7.53 × 10^7^ cell·mL^−1^) under the 0.04% CO_2_ (Fig. 2a). The number of *hct53* increased to 1.97 × 10^8^ cell·mL^−1^ under 5% CO_2_, 2.4 times higher than that of WT (8.3 × 10^7^ cell·mL^−1^; Fig. 2b).

As nitrogen depletion is a common practice to induce lipid production, we further quantified the growth performance of the mutant under the nitrogen-depleted condition (−N), both mutant and WT were cultured with columns. The difference between the mutant and WT strains was further aggravated under −N conditions, where the cell density of *hct53* was double that of WT under 0.04% CO_2_ (Fig. 2c) and almost tripled under 5% CO_2_ (Fig. 2d). In addition, we observed variations in cell size (Additional file 1: Figure S2a) and pigment content (Additional file 1: Figure S2b) of the mutants, indicating perturbations on endogenous metabolism by EMS mutagenesis. Meanwhile, a relatively similar pH value between the mutant and WT was observed under both +N and −N conditions with either 0.04% or 5% CO_2_ (Additional file 1: Figure S1). Overall, the *hct53* mutant shows a high-CO_2_-tolerance and produce more biomass than the parent strain under the high CO_2_ levels (i.e., 5%; Fig. 2). To better understand these changes, we examine the dynamics of primary metabolites in the microalgae.

### The *hct53* mutant is defective in starch biosynthesis and produces high amount of unsaturated fatty acids

**Fig. 3:**
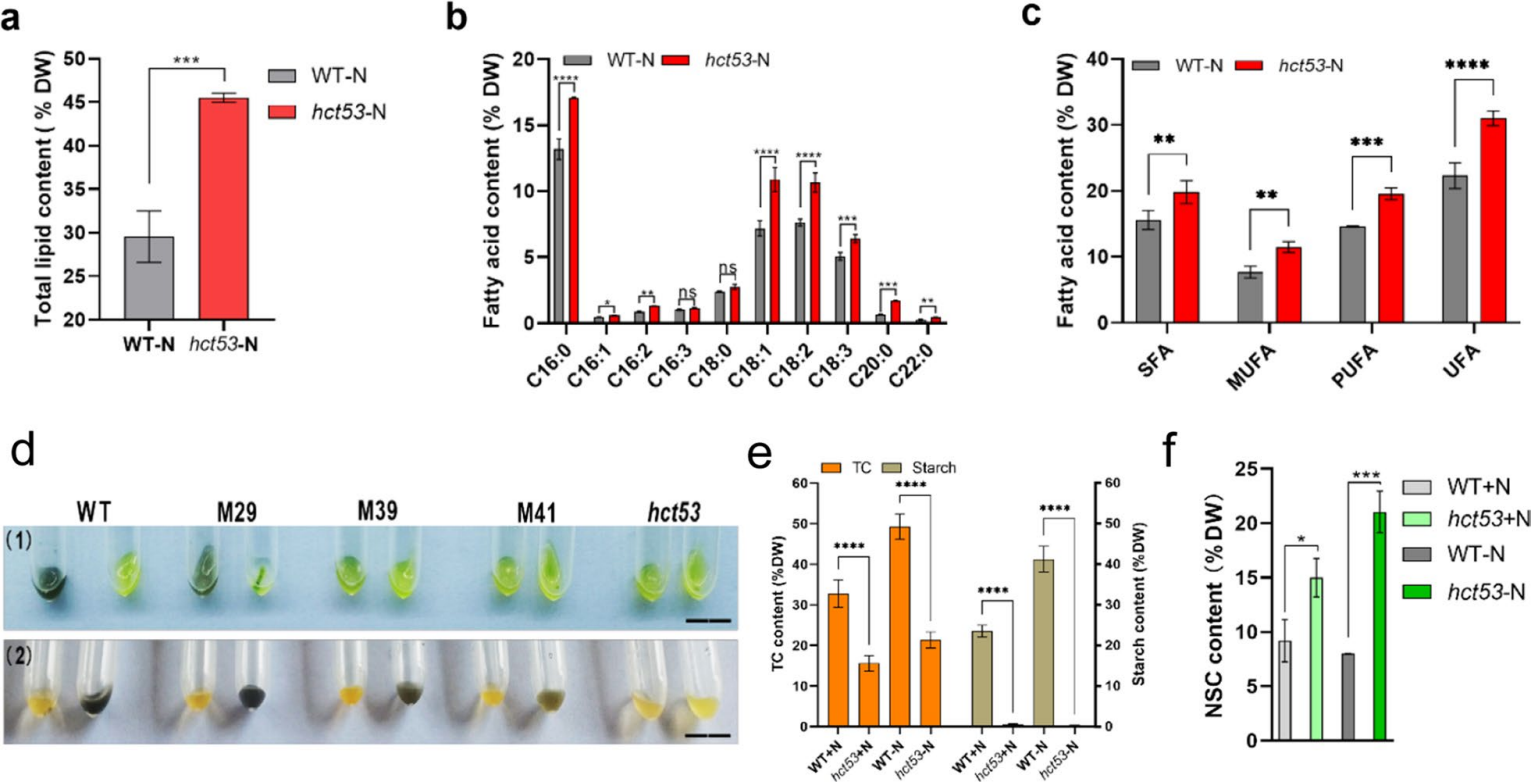
Metabolic comparison between WT and *hct53* raised with 5% CO_2_ aeration. **a** Comparison of total lipid content (% DW) between WT and *hct53*; **b** Comparison of individual fatty acid content (% DW) between WT and *hct53*; **c** Comparison of Lipid profiles (% DW) between WT and *hct53*. **d** Iodine vapor experiments of WT and the *hct53* mutant. Scale bar = 1.5 cm. **e** Comparison of total carbohydrate and starch content between WT and *hct53*; **b** Comparison of non-starch carbohydrate content between WT and *hct53*. SFA, saturated fatty acid; MUFA, monounsaturated fatty acid; PUFA, polyunsaturated fatty acid; UFA, unsaturated fatty acid; TC, total carbohydrate; NSC, non-starch carbohydrate; ns, no significant difference. *p < 0.0332; **p < 0.0021; ***p < 0.0002; ****p < 0.0001. Values represent means ± SD (*n* = 3)

To investigate the allocation of carbon flow, we examine the content of three primary metabolites in *hct53*. We found that total lipids in *hct53* increased significantly compared to the WT strain under −N and high-light conditions with 5% CO_2_ (from 29.55% to 45.51% DW; Fig. 3a). Moreover, the total content of unsaturated fatty acid (UFA; e.g., C16:1, C16:2, C16:3, C18:1, C18:2, and C18:3) was also increased from 22.3% to 31.0% DW (Fig. 3b, c).

We hypothesized that the increase in lipid production might be due to a blockage in starch biosynthesis, as lipids and starch share the same C3 metabolic precursors in microalgae. Iodine vapor experiments confirmed the hypothesis as that a bare level of starch in the *hct53* mutant (Fig. 3d). Under 5% CO_2_ aeration, the starch content in the WT strain in +N medium was 23.56%, which was elevated to 41.3% under the −N and high-light conditions (Fig. 3e). In contrast, no starch was observed in *hct53* mutant under both conditions (Fig. 3e). However, we still detected a considerable amount of non-starch carbohydrates (NSCs) in *hct53* under either condition, suggesting an active biosynthesis of NSCs in the mutant. Interestingly, *hct53* produced 62.93% more NSC than WT (9.2%; Fig. 3f) in the +N conditions, while the difference further increased to 262% under −N and high-light conditions (21.04% in *hct53*; Fig. 3f). Therefore, despite a blockage of starch biosynthesis, a shift of carbon flow to NSCs occurred in *hct53*.

### The *hct53* mutant shows high levels of SPs and high antioxidant capacity

**Fig. 4:**
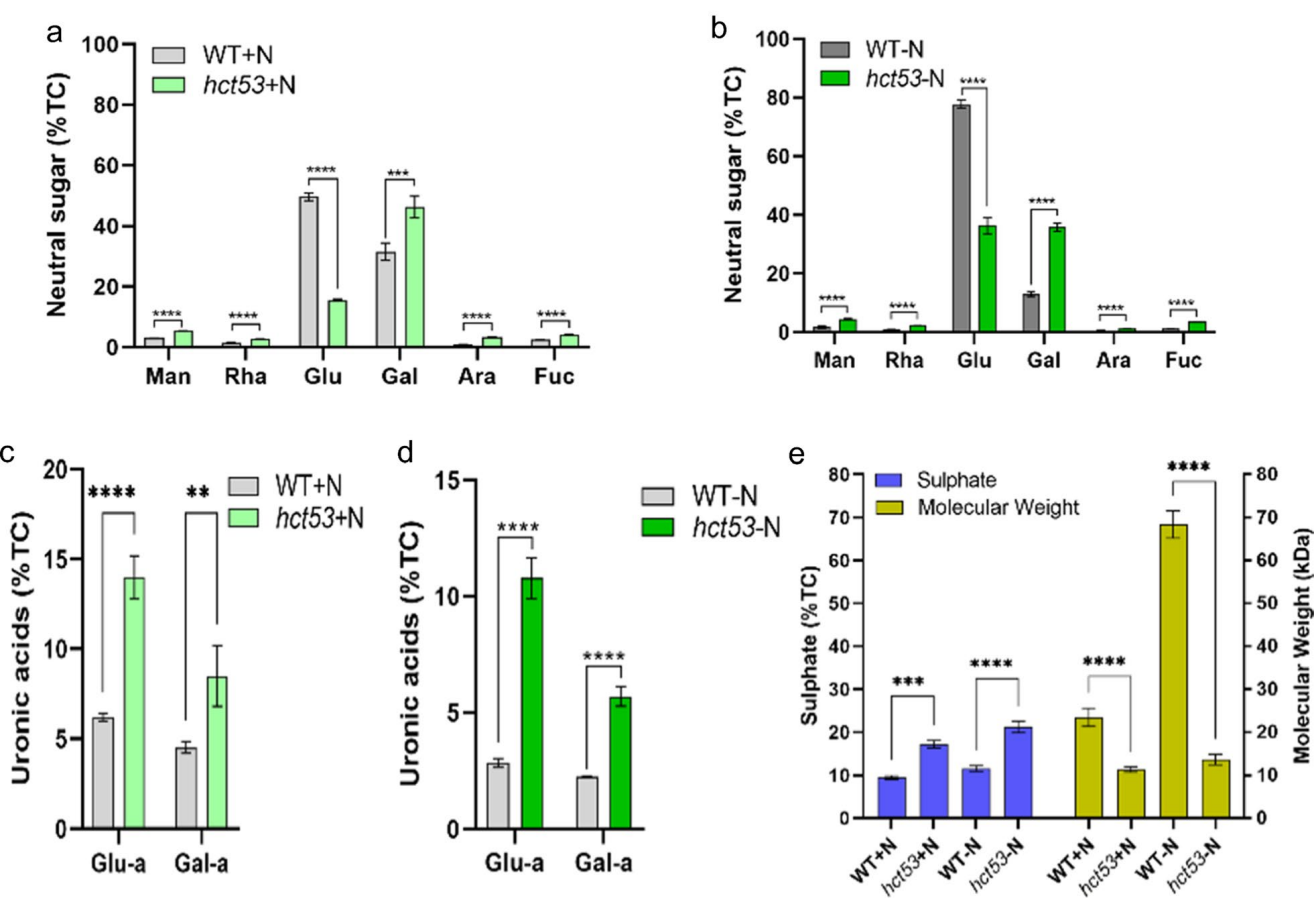
Saccharide profiles of *hct53* raised with 5% CO_2_ aeration. **a** Comparison of monosaccharide content between WT and *hct53* under +N conditions; **b** comparison of monosaccharide content between WT and *hct53* under −N conditions; **c** comparison of uronic acid content between WT and *hct53* under +N conditions; **d** comparison of uronic acid content between WT and *hct53* under −N conditions; **e** comparison of sulfate content and molecular weight between WT and *hct53*. +N, nitrogen repletion; −N, nitrogen depletion; Man, d-mannose; Rha, l-rhamnose; Glu-a, glucuronic acid; Gal-a, galacturonic acid; Glu, glucose; Gal, galactose, Ara, l-arabinose, Fuc, L-fucose. **p < 0.005; ***p < 0.0005; ****p < 0.0001. Values represent means ± SD (*n* = 3)

Compared to the WT strain, *hct53* showed increased levels of mannose, rhamnose, galactose, arabinose, and fucose under either +N (Fig. 4a) or the −N and high-light conditions (Fig. 4b), suggesting the presence of genetic switches controlling carbon towards specific monosaccharides. This finding is consistent with our previous research on *Chlorella sorokiniana* starchless mutant SLM3, where we observed an increase in specific monosaccharides, such as mannose [24].

FTIR analysis revealed that NSCs in *hct53* contain more functional sulfate groups than in WT, as indicated by the lower absorption value of the S=O stretching vibration of the sulfate group (see FTIR analysis in Additional file 1: Figure S3). Consistently, *hct53* produced higher amounts of polysaccharides containing uronic acids (i.e., glucuronic acid and galacturonic acid; Fig. 4c, d) and sulfate groups (Fig. 4e), which are known to have unique bioactivities and are widely used in medical and food industries [33, 34].

**Fig. 5:**
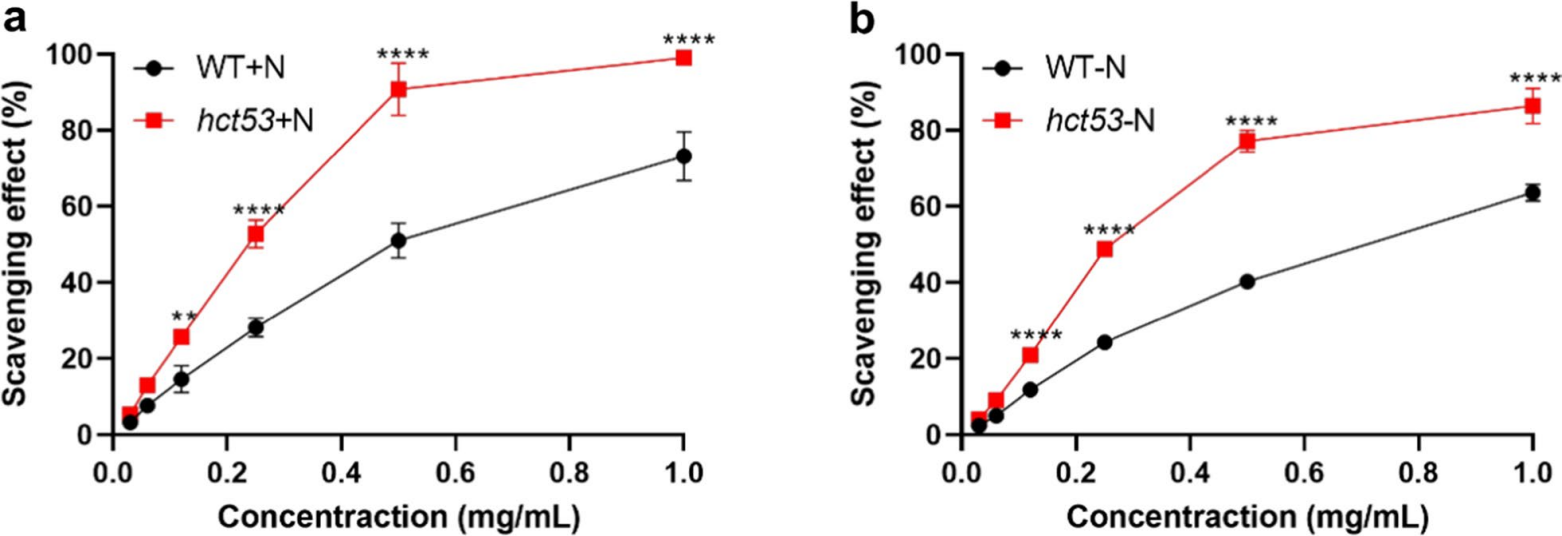
Scavenging capacity of *hct53* raised with 5% CO_2_ aeration. **a** Scavenging comparison between WT and *hct53* under +N conditions; **b** scavenging comparison between WT and *hct53* under -N conditions. +N, nitrogen repletion; −N, nitrogen depletion. **p < 0.005; ****p < 0.0001. Values represent means ± SD (*n* = 3). The log-phase microalgae cultured under 5% CO_2_ aeration were collected and used for scavenging capacity measurement

Furthermore, the molecular weights of total carbohydrates in *hct53* (11.32 kDa under the +N conditions, 13.67 kDa under the −N conditions) were 48.2% and 19.9% of that in WT under the counterpart conditions (Fig. 4e). In principle, the lower the molecular weights are, the higher the antioxidant activity of carbohydrates is [35]. Therefore, we suspect a higher antioxidant activity of *hct53* than that of WT. Indeed, *hct53* showed significantly higher DPPH scavenging capacity than WT under both conditions (Fig. 5a, b), indicating its potential as a source of antioxidant metabolites for use as food additives.

### The phenotypic shifts of the *hct53* mutant are underpinned by transcriptional dynamics

To dissect the molecular mechanisms underlying the robust CO_2_ fixation and metabolic shifts observed in *hct53*, the global gene expression in both WT and the mutant under the +N and high-light conditions was measured by mRNA-Seq, with three biological replicates for each sample (Additional file 2: Table S1; accession PRJNA892049). In total, over 38 million reads with an average length of 250 bp were produced for each sample, and 25,646 UniGenes (with an N50 length of 1834 bp and a GC content of 56.62%) were obtained after eliminating redundancy (Additional file 2: Table S2). PCA analysis supported a well-designed biological replication of WT and *hct53* (Additional file 1: Figure S4a).

Based on the definition of differential gene expression (see Methods), 1783 genes (56.69% of total) were found to be significantly upregulated, while 1362 genes (43.31% of total) were downregulated in *hct53* compared to WT (Additional file 1: Figure S4b). The downregulated genes were involved in various physiological functions without significant functional enrichment, while the upregulated genes showed significant functional enrichment in glycolysis, nitrogen metabolism, photosynthesis, citrate cycle (TCA cycle), peroxisome, and amino acid metabolism (Additional file 1: Figure S4c).

KEGG analysis identified the top 20 enriched metabolic pathways, which were divided into four functional aspects: metabolism of carbohydrates, amino acids, fatty acids, and other mixed metabolites. These results suggest that these metabolic pathways are closely related to the molecular mechanism engendering the properties of *hct53* (Additional file 1: Figure S4c).

#### Sources of carbon precursors

**Fig. 6:**
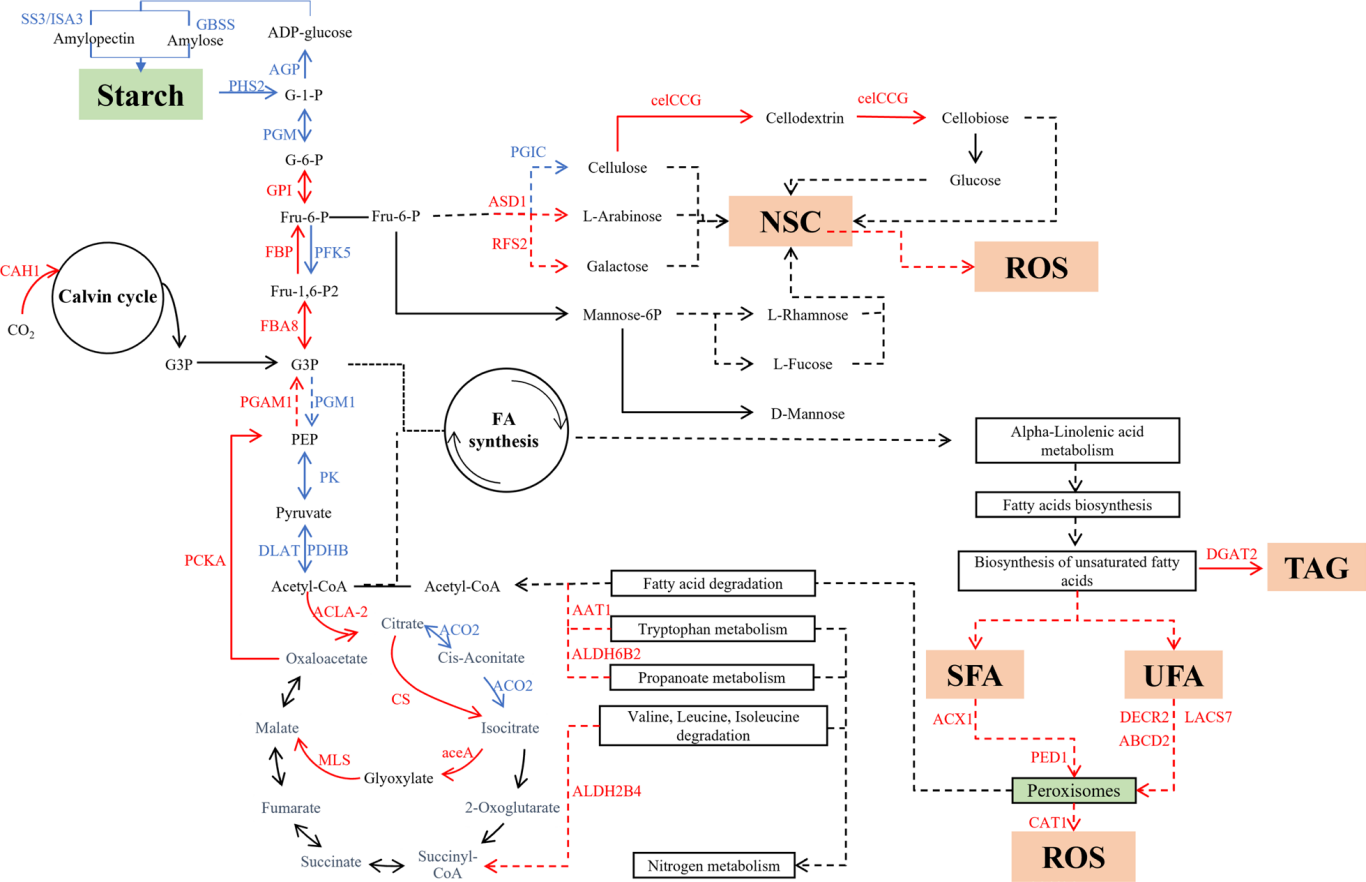
Schematic of molecular mechanism of *hct53*. Red font represents upregulated genes; blue font represents downregulated genes. Solid lines represent direct chemical reactions; dashed lines represent multistep chemical reactions. PEP, phosphoenolpyruvate; G3P, glycerol-3-phosphate; Fru-1,6-P2, fructose-1, 6-phosphate; Fru-6-P, fructose-6-phosphate; G-6-P, glucose-6-phosphate; G-1-P, glucose-1-phosphate; mannose-6P, mannose-6-phosphate; NPC, non-starch carbohydrate; ROS, reactive oxygen species; SFA, saturated fatty acid; UFA, unsaturated fatty acid, CAH1, Carbonic anhydrase CAH230; FBA8, fructose-1,6-bisphosphate aldolase; FBP, fructose-1,6-bisphosphatase I; PFK5, phosphofructokinase family; GPI, glucose-6-phosphate isomerase-like; PGIC, cytosolic phosphoglucose isomerase isoform A; PGM, phosphoglucomutase; AGP, glucose-1-phosphate adenylyltransferase large subunit, chloroplastic/amyloplastic; SS3, soluble starch synthase, chloroplastic/amyloplastic isoform A; GBSS, granule-bound starch synthase; ISA3, chloroplastic isoamylase; PHS2, cytosolic alpha-glucan phosphorylase; celCCG, endoglucanase A; ASD1, alpha-l-arabinofuranosidase 1 isoform B; RFS2, putative galactinol-sucrose galactosyltransferase 2; PGAM1, 2,3-bisphosphoglycerate-dependent phosphoglycerate mutase; PGM1, 2,3-bisphosphoglycerate-independent phosphoglycerate mutase; PK, pyruvate kinase isoform A; PDHB, pyruvate dehydrogenase E1 component; DLAT, pyruvate dehydrogenase E2 component; PCK, phosphoenolpyruvate carboxykinase; ACLA-2, ATP-citrate lyase A-2; CS, citrate synthase; MDH2, malate dehydrogenase; MLS, malate synthase; aceA, isocitrate lyase; ACO, aconitate hydratase; AAT1, acetyl-cytosolic 2; ALDH6B2, methylmalonate–semialdehyde dehydrogenase; ALDH2B4, aldehyde dehydrogenase; DGAT2, diacylglycerol acyltransferase; ACX1, acyl-CoA oxidase; PED1, peroxisomal 3-ketoacyl-thiolase; DECR2, 2,4-dienoyl-CoA reductase; LACS7, peroxisomal long-chain acyl synthetase; ABCD2, ABC transporter D family; CAT1, catalase isozyme 1

Carbonic anhydrases (CAs) catalyze the committed step in carbon dioxide concentration [36]. In the MEM25 genome, 13 CAs have been identified and classified into four categories: I (CP10g8552, CP1g691, and CP6g5531), II (CP10g8243 and CP2g2403), III (CP15g9947, CP4g3716, CP4g3717, CP5g5169, CP6g5615, and CP6g5616), and IV (CP10g8579 and CP8g6498; Additional file 1: Figure S5). Among them, CAH230 (CP4g3717) was significantly upregulated in *hct53* (Fig. 6; Additional file 2: Table S3), suggesting a potential more active CO_2_ sequestration in *hct53* cells than that of WT under the 5% CO_2_ conditions.

Light-harvesting complexes (LHCs) are generally used to harvest sunlight and transfer excitation energy to the reaction centers to drive photosynthesis. Green plants, including green algae, possess two distinct types of functional peripheral antenna complexes: Chl *a*-binding polypeptides (LHCAs associated with PSI) and Chl *b*-containing LHCs (LHCBs associated with PSII) [37]. Along with the increased CO_2_ capture, the transcripts of several LHCs (e.g., LHCA5, LHCB4, and LHCB5) were increased in the *hct53* mutant (Additional file 2: Table S3), suggesting a coordination between photosynthesis and CO_2_ concentration that simultaneously contributes to increased carbon fixation efficiency and biomass production in *hct53*.

#### Carbon partitioning diverting from starch and protein to lipids biosynthesis

Glyceraldehyde-3-phosphate (G3P), a key product of the Calvin cycle, is converted to pyruvate, the precursor for FA biosynthesis, via glycolysis pathways (Fig. 6). Phosophoglycerate mutase (PGAM) catalyzes the reversible conversion of 3-phosphoglycerate (3-PG) and 2-phosphoglycerate (2-PG) during the process of glycolysis. The *PGM* gene was dramatically increased in a starch-rich *Chlamydomonas reinhardtii* mutant [38], while PGM involved in the metabolism of glucose decrease in animals [39], suggesting that PGM could play a role in stimulating the carbon flow from glucose to starch. The *PGM* transcripts (CP4g4298) depressed dramatically in *hct53*, which is compatible with the compromised starch biosynthesis (Fig. 6; Additional file 2: Table S3). The downregulation of *PGM* gene was concomitant with the increase in the transcripts of genes responsible for glycolysis, such as glucose-6-phosphate isomerase (G-6-P, CP1g370) and fructose-bisphosphate aldolase (FBA, CP13g9181; Fig. 6; Additional file 2: Table S3), suggesting an elevated level of glycolysis. Fructose-1,6-bisphosphatase (FBP), a rate-limiting enzyme in gluconeogenesis, catalyzes the irreversible splitting of fructose 1,6-diphosphate to fructose-6-phosphate. FBP1 deficiency impairs the formation of glucose from lactate, glycerol, and gluconeogenic amino acids, such as alanine.

FBP (CP11g7978) was transcriptional elevated in *hct53* (Fig. 6; Additional file 2: Table S3), suggesting a rebalance of carbon flow to glucose via gluconeogenesis. Meanwhile, the transcript of the gene encoding phosphoenolpyruvate carboxykinase (PCK, CP3g2830), a critical enzyme in gluconeogenesis (whose enhanced activity leads to increased glucose output), was also increased in the mutant (Fig. 6; Additional file 2: Table S3). Therefore, the global carbon flow in the mutant could be interpreted as follows: a depressed synthesis of starch biosynthesis and an increased activity of gluconeogenesis cooperatively lead to an increased amount of glucose in the mutant, while the generated glucose is broken down via elevated glycolysis into more pyruvate and ATP.

Moreover, genes encoding the enzymes involved in the catabolic pathways of tryptophan (acetyl-cytosolic 2, AAT1; CP3g3167), propanoate (methylmalonate–semialdehyde dehydrogenase, ALDH6B2; CP11g7986), valine, leucine, and isoleucine (aldehyde dehydrogenase, ALDH2B4; CP14g9609) were also activated (Fig. 6; Additional file 2: Table S3), suggesting an elevated activity of amino acid degradation and thus a deviation of carbon flow from the amino acids, in agreement with the decreased protein levels in the mutant.

The building block for lipid biosynthesis is acetyl-CoA which is generated from pyruvate or the citrate cycle (TCA cycle). A number of genes involved in citrate cycle were transcriptionally activated, such as the genes encoding the enzymes catalyzing the sequential conversion from malate to oxaloacetate (malate dehydrogenase, MDH2; CP14g9476), citrate (citrate synthase, CS; CP8g6223), and acetyl-CoA (ATP-citrate lyase A-2, ACLA-2; CP13g9081; Fig. 6; Additional file 2: Table S3). In contrast, aconitate hydratase (ACO2, CP14g9383; the second enzyme of the citrate cycle), catalyzing the isomerization of citrate to isocitrate, is depressed in the mutant (Fig. 6; Additional file 2: Table S3), suggesting a potential blockage of the conversion from citrate to isocitrate and thus an increased accumulation of citrate for acetyl-CoA production.

Acyl-CoA:diacylglycerol acyltransferase (DGAT, EC 2.3.1.20) catalyzes the last reaction in the acyl-CoA-dependent biosynthesis of triacylglycerol (TAG). In the MEM25 genome, two DGAT genes (CP11g7967 and CP11g7969) were identified. Along with the increased levels of genes relating to acetyl-CoA biosynthesis, a simultaneous elevation of the transcript of CP11g7969 was observed in the mutant (Fig. 6; Additional file 2: Table S3), suggesting that these genes may contribute to the increased level of TAG in the mutant. Meanwhile, transcripts of genes relating to glyoxylate cycle, such as isocitrate lyase (*aceA*, CP3g3555) and malate synthase (MLS, CP3g2923), were increased in the mutant (Fig. 6; Additional file 2: Table S3), which potentially contribute to biomass generation by conserving carbon skeletons via bypassing the oxidative decarboxylation steps of the citrate cycle [40]. Therefore, the choreography of the transcripts was concomitant with the increase in lipid synthesis and the decrease in the synthesis of starch and proteins in the mutant, suggesting that these genes are responsible for carbon shift from sugars and proteins to lipid synthesis.

#### Elevated accumulation of NSCs and antioxidant enzymes contribute to the high antioxidant capacity

The occurrence of more NSCs in the carbohydrate pool in *hct53* suggests the presence of genetic switches controlling the precise carbon allocation to specific monosaccharides. In *hct53*, genes encoding enzymes involved in the biosynthesis of specific monosaccharides were elevated, such as α-l-arabinofuranosidase 1 (involved in l-arabinose biosynthesis; ASD1, CP1g68) and galactinol-sucrose galactosyltransferase (involved in galactose; RFS2, CP1g456; Fig. 6; Additional file 2: Table S3). This is consistent with the increased accumulation of galactose and arabinose (Fig. 4a, b). Meanwhile, genes encoding enzymes related to the antioxidant system in peroxisomes, such as catalase isozyme 1 (CAT1, CP6g5547) was upregulated (Fig. 6; Additional file 2: Table S3). Together with the elevated biosynthesis of SPs (Fig. 4e), these transcriptional and metabolic alterations underpin the higher antioxidant capacity of *hct53* compared to the WT strain.

### The transcriptional dynamics in the *hct53* mutant is engendered by the genomic mutations

To shed light on the genomic basis of the transcriptional dynamics of *hct53*, we probed the pattern and frequency of introduced mutations at a genome-level. In total, we confirmed 207 mutant sites in the *hct53* mutant, which potentially resulted in mutation in 392 genes (Additional file 1: Figure S6a; Additional file 2: Table S4). EMS-mediated mutagenesis predominantly results in a transition from guanine (G) to adenine (A) (99% of mutations) in higher plants [41]. In MEM25, transitions from G to A were most frequent (23.19%), followed by transitions from cytosine (C) to thymine (T) (16.43%) (Additional file 1: Figure S6a). To examine whether and how the mutated genes alter carbon partitioning in the mutant, we conducted an in-depth investigation on the mutated genes.

To characterize the “hotspots” and “coldspots” of EMS-induced single nucleotide polymorphisms (SNPs), we categorized the genomic regions of each gene into upstream (i.e., 1 kilobase (kb) upstream of the most distal transcription start site), 5′-untranslated regions (UTRs), exons, introns, 3′-UTRs, and downstream regions (i.e., 1 kb downstream of the most distal polyadenylation site; Additional file 1: Figure S6b). For the introns, we specify the splice regions (i.e., splice donors and splice acceptors) which could influence the final amino acid sequences of the proteins and thereby the function.

The largest number of SNPs in the *hct53* mutant are in downstream regions (152) followed by those in upstream regions (135), introns (103), intergenic regions (62), splice regions (46), exons (40), 3′-UTRs (4), and 5′-UTRs (0) (Additional file 1: Figure S6b; Additional file 2: Table S4). Among the mutations in exons, the number of missense, stop-gained, frameshift, and stop-lost variants are 24, 3, 1, and 1, respectively (Additional file 1: Figure S6; Additional file 2: Table S4). Numerous of mutations are relating to the process of TCA cycle or providing sources of carbon precursors. Specifically, a frameshift occurs in low-CO_2_ inducible protein (CP1g18; Additional file 2: Table S4), which may contribute the high-CO_2_ tolerance of the *hct53* mutant. Moreover, a missense mutation occurs in a gene encoding alcohol dehydrogenase (ADH; CP3g2759; Additional file 2: Table S4) which catalyzes the oxidation of ethanol into acetaldehyde and subsequently acetate.

Consistent with the overall decreased content of terpenoids (such as chlorophylls and carotenoids), genes encoding the committed enzymes in the methylerythritol 4-phosphate (MEP) pathway harbor mutations either in the exon (acetyl-CoA C-acetyltransferase, CP3g3167) or the upstream regions (squalene synthase, CP15g9782; Additional file 2: Table S4). In particular, mutations have been detected in genes involved in chlorophyll biosynthesis (e.g., porphobilinogen deaminase CP10g8503 and chlorophyllide a oxygenase CP8g6404) and carotenogenesis (ζ-carotene isomerase; CP9g7254 and CP6g5456; Additional file 2: Table S4), consistent with the low levels of chlorophylls and carotenoids in *hct53* (Additional file 1: Figure S2b).

We also observed mutations involved in chloroplast development (i.e., pentatricopeptide repeat domain-containing protein 1, CP16g10093) and photosynthesis, such as light-harvesting protein (CP12g8969) and low PSII Accumulation 3 protein (CP1g972; involves in photosystem II assembly; Additional file 2: Table S4). However, the mutations occur either in upstream regions or introns, where the consequence of these mutations remains to be validated.

In the mutant, carbon partition is diverted from starch and protein to lipids. Coincidently, the mutations are significantly enriched in genes relating to gluconeogenesis and glycolysis, including 1,3-β-glucan synthase (CP7g6853; which plays major roles in polysaccharide synthesis), phosphoenolpyruvate carboxykinase (CP7g6893; a critical enzyme in gluconeogenesis), glucose-6-phosphate 1-epimerase (CP3g3128; which participates in glycolysis/gluconeogenesis), pyruvate kinase (CP3g3075; which catalyzes the irreversible conversion of ADP and phosphoenolpyruvate to ATP and pyruvic acid in the last step of glycolysis), phosphofructokinase family isoform B (CP1g878; involved in glycolysis), glyoxalase (CP1g875; a focal point in glycolysis), pyrophosphate-fructose 6-phosphate 1-phosphotransferase (CP1g742; which regulates starch biosynthesis), glycerol kinase (CP1g612; which phosphorylates glycerol forming glycerol 3-phosphate), and fructose-1,6-bisphosphate aldolase (CP1g1128; which plays central roles in glycolysis and gluconeogenesis) and protein synthesis, such as subunit ribosomal protein (e.g., CP3g3313 and CP5g4677), ubiquitin-conjugating enzymes (e.g., CP9g7669 and CP10g8242), 26S proteasome (e.g., CP1g908), oligopeptidase (e.g., CP11g7848), and genes relating to transfer RNA (tRNA; e.g., CP11g8025 and CP10g8241; Additional file 2: Table S4).

Apart from mutations in saccharometabolism and protein-synthetic genes, a number of genes with the putative function of recycling fatty acids (FAs) from membrane lipids for TAG synthesis were found to be mutated. These include lipase (i.e., sn1-specific diacylglycerol lipase beta CP5g5110, sn1-specific diacylglycerol lipase beta CP5g5111, and phospholipase A I-like CP16g10067), glycerol-3-phosphate acyltransferase 3-like (i.e., CP1g1534; the rate-limiting enzyme in the de novo pathway of glycerolipid synthesis), cyclopropane-fatty-acyl-phospholipid synthase (i.e., CP5g4531 and CP2g2312; regulating the levels of cyclopropane fatty acids), and choline-phosphate cytidylyltransferase (i.e., CP5g5046 and CP5g5047; catalyzing a rate-limiting step in the CDP–choline pathway for the synthesis of phosphatidylcholine and phosphatidylcholine-derived lipids; Additional file 2: Table S4). In addition, a mutation has been detected in the intron region of the only Acyl-coenzyme A oxidase (ACO) gene (i.e., CP5g4861) identified in the MEM25 genome. ACO is the rate-limiting enzyme that catalyzes the initial step of the β-oxidation system in the peroxisome.

Despite the decrease in starch content, the *hct53* mutant showed an increase in the content of several sugars, including mannose and raffinose. Mutations were found in the downstream region of genes encoding mannose-P-dolichol utilization defect 1-like protein (CP4g4195; which is required for utilization of the mannose donor mannose-P-dolichol in the synthesis of lipid-linked oligosaccharides and glycosylphosphatidylinositols) and raffinose synthase (CP3g3064; which catalyzes raffinose formation; Additional file 2: Table S4). This suggests that these genes may act as genetic switches controlling the allocation of carbon to specific monosaccharides, although their specific roles remain unclear. In addition, mutations were identified in genes involved in antioxidant processes, such as glutathione peroxidase CP12g8697, thioredoxin CP7g6895, and phospholipid–hydroperoxide glutathione peroxidase CP3g2818. These mutations were mostly found in up- or downstream regions (Additional file 2: Table S4), which may contribute to the high antioxidant capacity observed in the *hct53* mutant (Fig. 5).

Overall, the genome-wide distribution of mutations and temporally differential expression may affect transcription or translation of crucial biological processes in the mutant. While the actual effects of these mutations need to be confirmed on a case-by-case basis, they collectively contribute to the phenotypic shifts observed in the *hct53* mutant, including increased lipid and non-structural carbohydrate synthesis, compromised protein and starch synthesis, and high CO_2_ tolerance.

## Conclusions

The *hct53* mutant of *Chlorella* MEM25 was generated by EMS mutagenesis and was found to have high CO_2_ tolerance. The mutant exhibited changes in carbon partitioning, with a shift from starch and protein biosynthesis to lipid and antioxidant synthesis. To understand the molecular mechanisms underlying these changes, genome-wide mutations and transcriptomic dynamics were studied in the mutant and parent strain under high CO_2_ conditions. At the transcript level, enhanced CO_2_ tolerance was linked to upregulation of putative genes related to photosynthesis and CO_2_ concentration. Transcriptional stimulation was observed in pathways that direct carbon precursors from protein and starch metabolic pathways towards glycerolipid synthesis. Specifically, genes involved in supplying carbon precursors and energy for de novo fatty acid synthesis, including those encoding components of the citrate cycle, triacylglycerol, and glyoxylate cycle, were upregulated.

The higher antioxidant capacity of the *hct53* mutant was also observed, which could contribute to the elevation of genes involved in the biosynthesis of specific monosaccharides, such as galactose and arabinose, and the antioxidant system, such as catalase. This choreography of transcripts was engendered by the genomic mutations, which togetherly contributed to the phenotypic shifts observed in the *hct53* mutant, including high CO_2_ tolerance, compromised protein and starch synthesis, and increased lipid synthesis. These findings have important implications for improving the beneficial properties of microalgae as functional food and for deciphering the molecular mechanisms underlying carbon flow from “redundant compounds” to synthesize valuable ones.

### Supplementary Information

**Additional file 1.** **Fig. S1. **Dynamics of pH values of WT and *hct53* culture under different conditions. (a) nitrogen-replete conditions aerated with 0.04% CO_2_; (b) nitrogen-repleted conditions aerated with 5% CO_2_; (c) nitrogen-depleted conditions aerated with 0.04% CO_2_; (d) nitrogen-depleted conditions aerated with 5% CO_2_. **Fig. S2.** Phenotype comparison between WT and *hct53* culture. (a) Cell size; (b) Pigment content. ***p < 0.0002; ****p < 0.0001. Values represent means ± SD (*n* = 3). **Fig. S3. **Fourier transform infrared spectroscopy of the S=O stretching vibration of the sulfate group in the non-starch carbohydrates. (a) +N conditions; (b) −N conditions. Abbreviations: +N, nitrogen-replete conditions; −N, nitrogen-depleted conditions. The absorption values of the S=O stretching vibration (1239 cm^−1^ and 1256 cm^−1^) correspond to the sulfate groups. **Fig. S4. **The principal component analysis (PCA) of transcriptionally altered genes and top 20 metabolic pathways in *hct53*. (a) PCA score plot; (b) Numbers of transcriptionally altered genes; (c) Scatter plot of the top 20 metabolic pathways. Note: Rich factor represents the number of differential genes located in KEGG and a greater Rich factor value indicates greater KEGG enrichment. Triangles, circles, diamonds, and rectangles represent metabolic pathways relating to amino acids, carbohydrates, lipids, and mixed metabolic pathways. **Fig. S5. **Phylogenetic and motif analysis of carbonic anhydrases (CAs). (a) Phylogenetic tree of CAs. Orthofinder was used to annotate multiple species homologous genes, where in MEM25, there are 13 CA-related homologs categorized into four groups (see main text for details). (b) Motifs of CAs. MEME was used for motifs identification. (c) Conserved domains of CAs. MEM25 CAs have four types of conversed domains, namely, cd00883: beta_CA_cladeA, cd03379: beta_CA_cladeD, cl33453: carbonate dehydratase and cd03124: alpha_CA_prokaryotic_like. **Fig. S6. **The pattern and frequency of introduced mutations *hct53* genome. (a) The mutation pattern and frequency; (b) the mutation regions; (c) the mutation types.

**Additional file 2.** **Table S1**. Overview of transcriptomic sequencing data sets. **Table S2**. Overview of Trinity assembly. **Table S3**. Transcriptomic comparison of transcriptionally altered key genes between WT and *hct53* under the high light and nitrogen-depleted conditions with 5% CO_2_. **Table S4**. Mutated genes in *hct53.*

## Data Availability

All data generated or analyzed during this study are included in this published article and its supplementary information files.
