# Correction: Artificial switches induce the bespoke production of functional compounds in marine microalgae *Chlorella* by neutralizing CO_2_

**DOI:** 10.1186/s13068-023-02423-y

**Published:** 2023-11-16

**Authors:** Jiahua Gu, Yuan Xiao, Mingcan Wu, Aoqi Wang, Xinyu Cui, Yi Xin, Kalyanee Paithoonrangsarid, Yandu Lu

**Affiliations:** 1 Single‑cell BioEngineering Group, State Key Laboratory of Marine Resource Utilization in South China Sea, School of Marine Biology and Fisheries, Hainan University, Haikou, 570228 China; 2 Biochemical Engineering and Systems Biology Research Group, National Center for Genetic Engineering and Biotechnology, National Science and Technology Development Agency, King Mongkut’s University of Technology Thonburi, Bangkok, Thailand; 3 Hainan Provincial Key Laboratory of Tropical Hydrobiotechnology, Hainan University, Haikou, China; 4 Haikou Technology Innovation Center for Research and Utilization of Algal Bioresources, Hainan University, Haikou, China

**Correction: Biotechnology for Biofuels and Bioproducts (2023) 16:143** **https://doi.org/10.1186/s13068-023-02381-5**

Following publication of the original article [1], the authors, “Jiahua Gu, Yuan Xiao, Mingcan Wu and Aoqi Wang” should have been denoted as equally contributing authors. This has now been corrected with this erratum.

The original article has been corrected.
