# Supplementary material for: Artificial switches induce the bespoke production of functional compounds in marine microalgae Chlorella by neutralizing CO2
Source: Biotechnol Biofuels Bioprod. 2023 Sep 27;16:143. doi: 10.1186/s13068-023-02381-5 (PMC10537470; doi:10.1186/s13068-023-02381-5)
Supplement: Supplementary file 1 — Additional file 1. Fig. S1. Dynamics of pH values of WT and hct53 culture under different conditions. (a) nitrogen-replete conditions aerated with 0.04% CO2; (b) nitrogen-repleted conditions aerated with 5% CO2; (c) nitrogen-depleted conditions aerated with 0.04% CO2; (d) nitrogen-depleted conditions aerated with 5% CO2. Fig. S2. Phenotype comparison between WT and hct53 culture. (a) Cell size; (b) Pigment content. ***p < 0.0002; ****p < 0.0001. Values represent means ± SD (n = 3). Fig. S3. Fourier transform infrared spectroscopy of the S=O stretching vibration of the sulfate group in the non-starch carbohydrates. (a) +N conditions; (b) −N conditions. Abbreviations: +N, nitrogen-replete conditions; −N, nitrogen-depleted conditions. The absorption values of the S=O stretching vibration (1239 cm−1 and 1256 cm−1) correspond to the sulfate groups. Fig. S4. The principal component analysis (PCA) of transcriptionally altered genes and top 20 metabolic pathways in hct53. (a) PCA score plot; (b) Numbers of transcriptionally altered genes; (c) Scatter plot of the top 20 metabolic pathways. Note: Rich factor represents the number of differential genes located in KEGG and a greater Rich factor value indicates greater KEGG enrichment. Triangles, circles, diamonds, and rectangles represent metabolic pathways relating to amino acids, carbohydrates, lipids, and mixed metabolic pathways. Fig. S5. Phylogenetic and motif analysis of carbonic anhydrases (CAs). (a) Phylogenetic tree of CAs. Orthofinder was used to annotate multiple species homologous genes, where in MEM25, there are 13 CA-related homologs categorized into four groups (see main text for details). (b) Motifs of CAs. MEME was used for motifs identification. (c) Conserved domains of CAs. MEM25 CAs have four types of conversed domains, namely, cd00883: beta_CA_cladeA, cd03379: beta_CA_cladeD, cl33453: carbonate dehydratase and cd03124: alpha_CA_prokaryotic_like. Fig. S6. The pattern and frequency of introduced mutations hct [file 13068_2023_2381_MOESM1_ESM.docx]

**Supplementary materials**

**
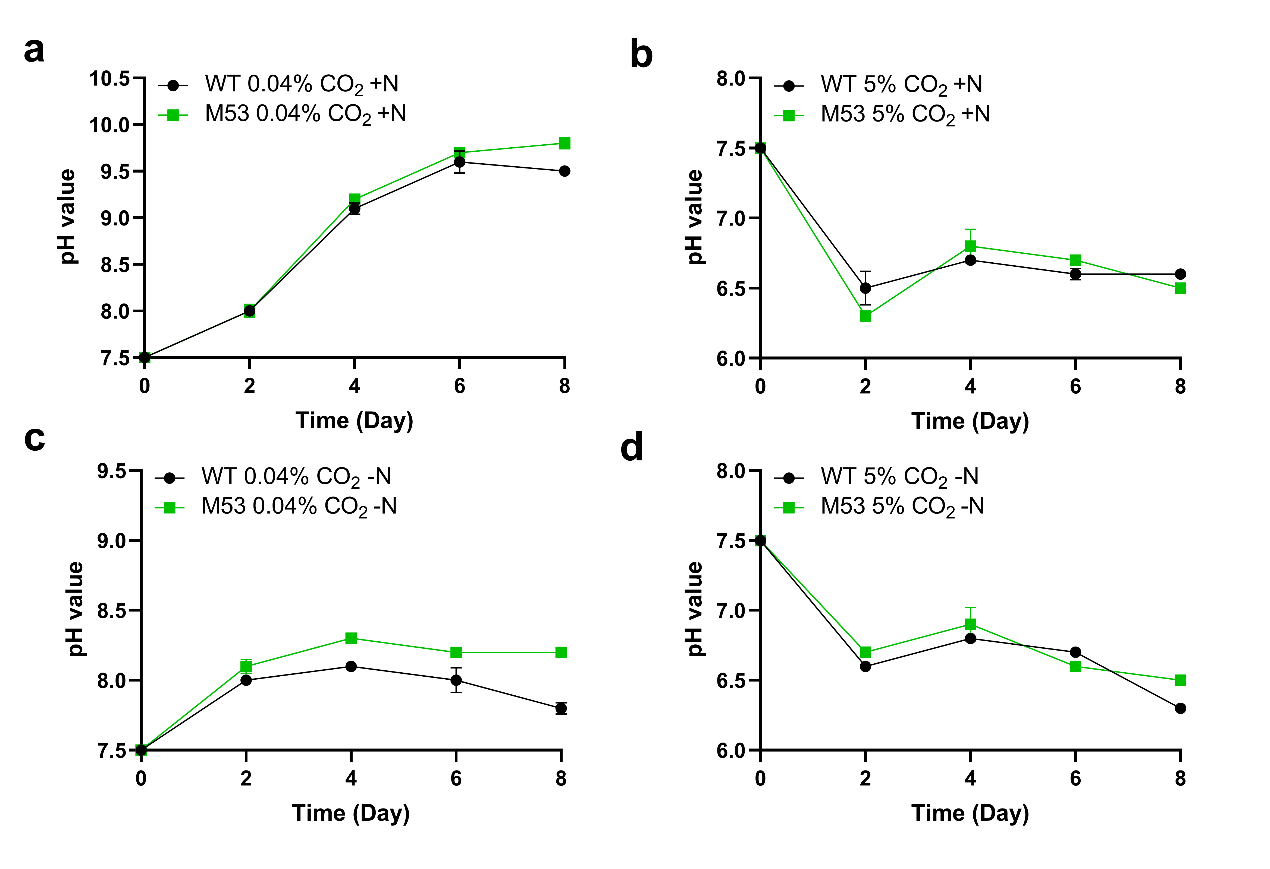
Fig. S1. Dynamics of pH values of WT and *hct53* culture under different conditions**. (a) nitrogen-replete conditions aerated with 0.04% CO_2_; (b) nitrogen-repleted conditions aerated with 5% CO_2_; (c) nitrogen-depleted conditions aerated with 0.04% CO_2_; (d) nitrogen-depleted conditions aerated with 5% CO_2_.

**
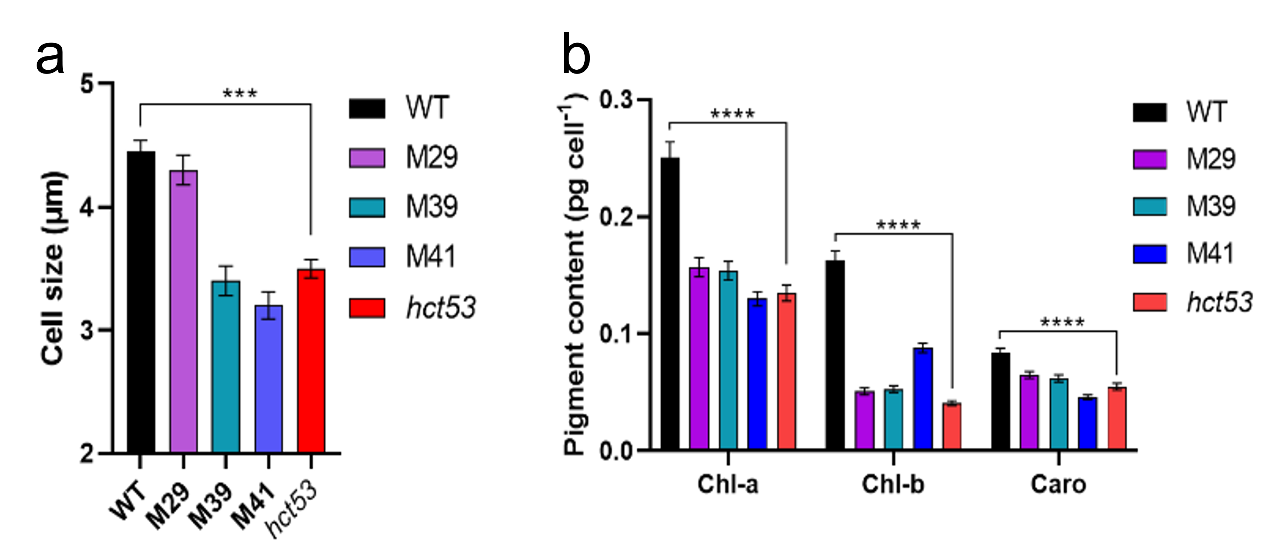
Fig. S2. Phenotype comparison between WT and *hct53* culture**. (a) Cell size; (b) Pigment content. ***, *p* < 0.0002; ****, *p* < 0.0001. Values represent means ± SD (n = 3).

**
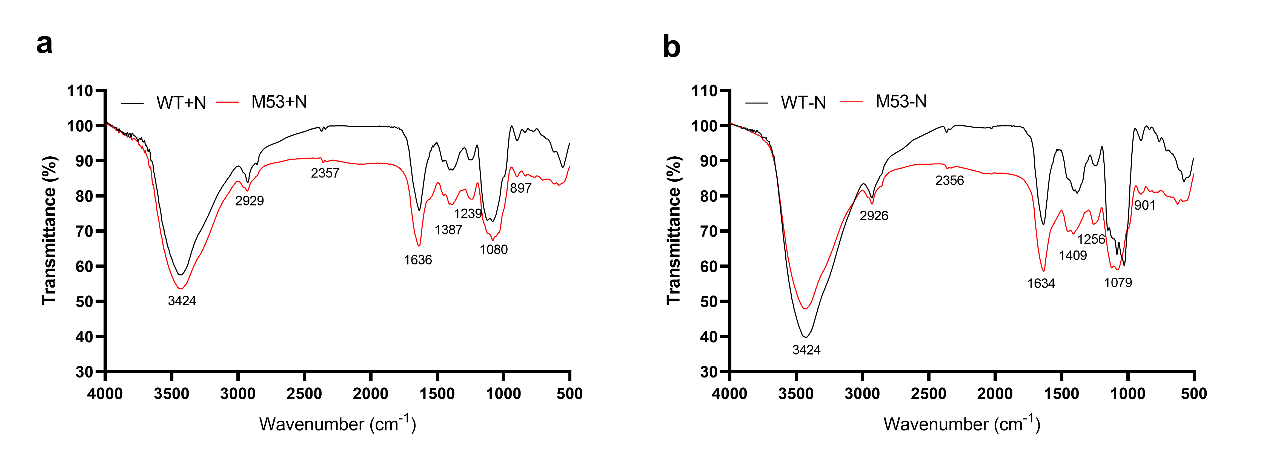
Fig. S3. Fourier transform infrared spectroscopy of the S=O stretching vibration of the sulfate group in the non-starch carbohydrates**. (a) +N conditions; (b) −N conditions. Abbreviations: +N, nitrogen-replete conditions; −N, nitrogen-depleted conditions. The absorption values of the S=O stretching vibration (1239 cm−1 and 1256 cm−1) correspond to the sulfate groups.

**
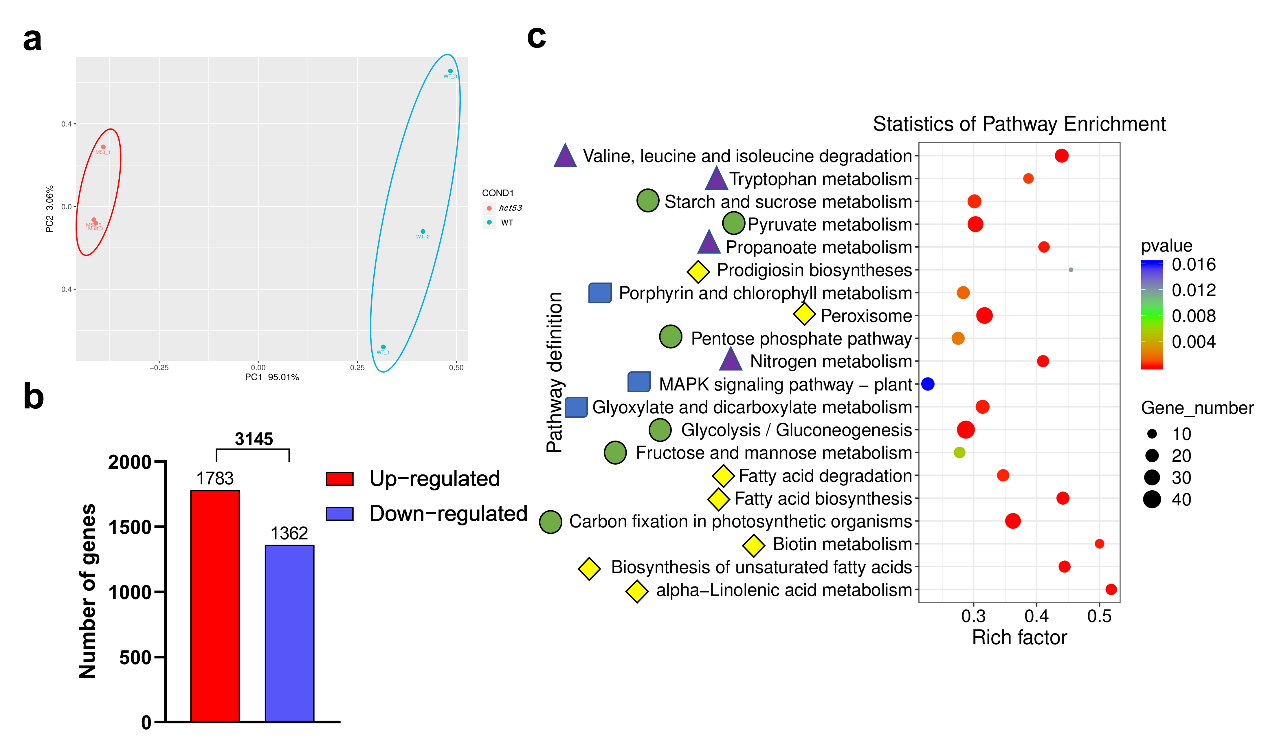
Fig. S4. The principal component analysis (PCA) of transcriptionally altered genes and top 20 metabolic pathways in *hct53***. (a) PCA score plot; (b) Numbers of transcriptionally altered genes; (c) Scatter plot of the top 20 metabolic pathways. Note: Rich factor represents the number of differential genes located in KEGG and a greater Rich factor value indicates greater KEGG enrichment. Triangles, circles, diamonds, and rectangles represent metabolic pathways relating to amino acids, carbohydrates, lipids, and mixed metabolic pathways.

**
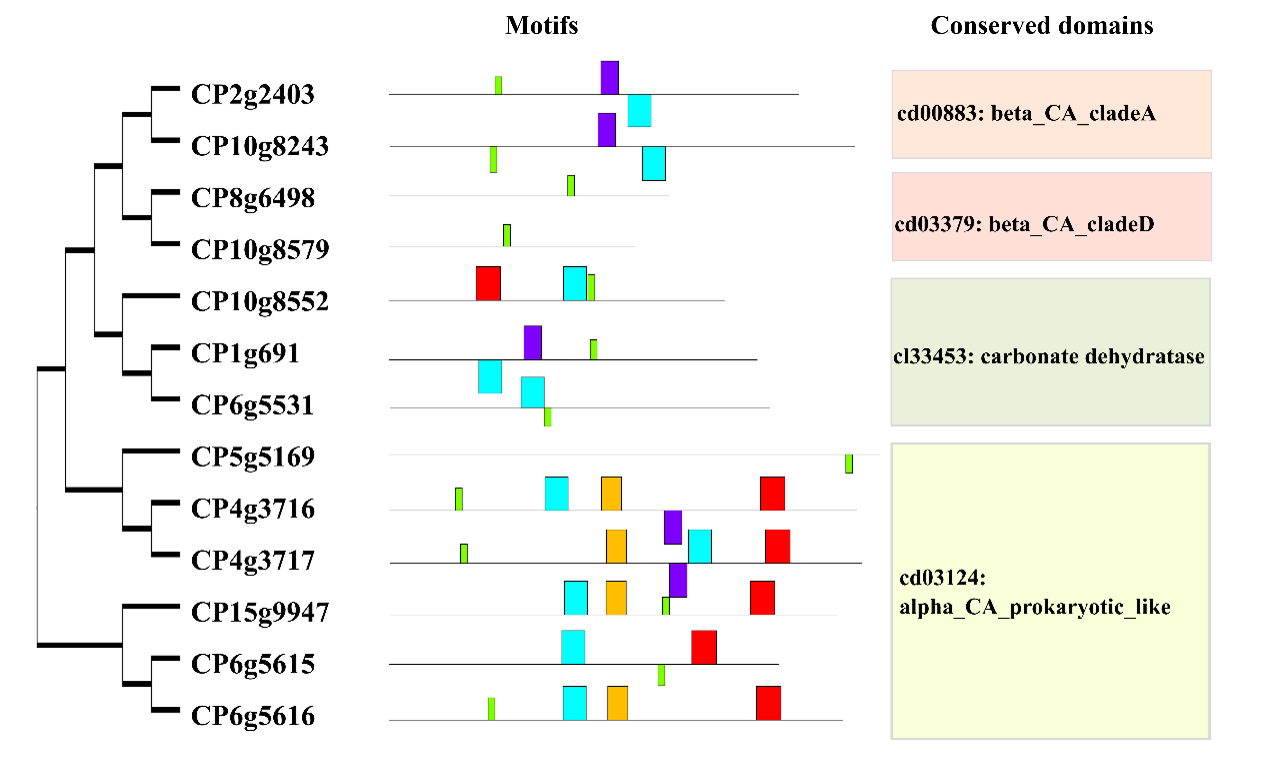
Fig. S5. Phylogenetic and motif analysis of carbonic anhydrases (CAs)**. (a) Phylogenetic tree of CAs. Orthofinder was used to annotate multiple species homologous genes where in MEM25, there are 13 CA-related homologs categorized into four groups (see main text for details). (b) Motifs of CAs. MEME was used for motifs identification. (c) Conserved domains of CAs. MEM25 CAs have four types of conversed domains, namely cd00883: beta_CA_cladeA, cd03379: beta_CA_cladeD, cl33453: carbonate dehydratase and cd03124: alpha_CA_prokaryotic_like.

**
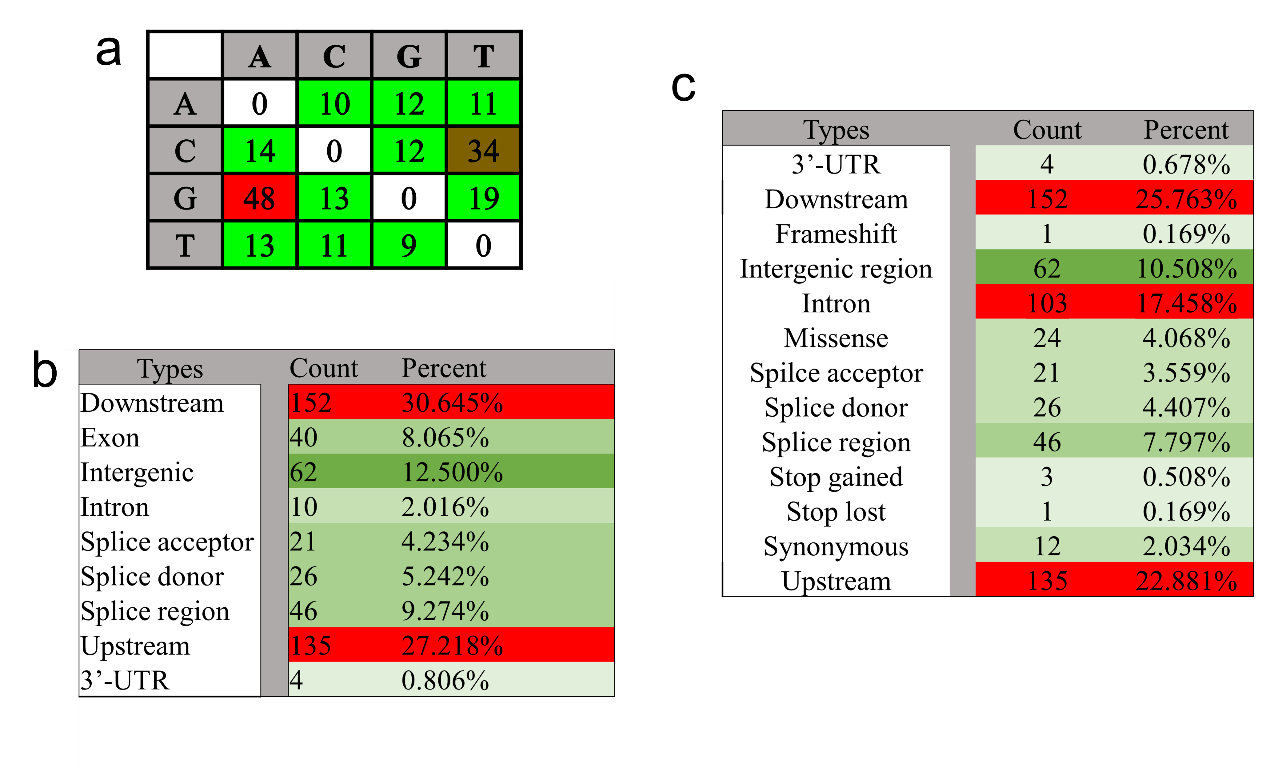
Fig. S6. The pattern and frequency of introduced mutations *hct53* genome**. (a) the mutation pattern and frequency; (b) the mutation regions; (c) the mutation types.
